# Recovery housing and on-the-ground research priorities: a scoping study through the lens of community based participatory research

**DOI:** 10.3389/fpubh.2025.1554344

**Published:** 2025-05-22

**Authors:** Patrick F. Hibbard, Cameron W. Tice, Jodie M. Dewey, Kathryn R. Gallardo, James Tompkins, Justin S. Bell, Jasleen Sandhu, Michelle A. Cruz, Kathryn Babbitt, Amy A. Mericle, Ashli J. Sheidow

**Affiliations:** ^1^Chestnut Health Systems, Lighthouse Institute, Bloomington, IL, United States; ^2^Department of Psychology, University of Cambridge, Cambridge, United Kingdom; ^3^Department of Health Promotion and Behavioral Sciences, UTHealth Houston School of Public Health, Houston, TX, United States; ^4^California State University, Bakersfield, CA, United States; ^5^DePaul University, Chicago, IL, United States; ^6^Department of Chemical Engineering and Biotechnology, University of Cambridge, Cambridge, United Kingdom; ^7^Public Health Institute, Alcohol Research Group, Emeryville, CA, United States

**Keywords:** recovery housing, community-based participatory research (CBPR), scoping study, recovery science, recovery support services, recovery residences

## Abstract

**Introduction:**

Though communities have featured recovery housing (RH) for several decades, the base of evidence for best practices continues to grow – especially evidence needed by, and known to, those who operate and receive these services. The Initiative for Justice and Emerging Adult Populations (JEAP) engaged with three community boards (CBs) – consisting of young adults with experience in recovery from substance use issues, people who have a history of criminal legal system involvement and recovery, and payers and provider of substance use services and harm reduction – to understand on-the-ground priorities for research into recovery support services.

**Methods:**

JEAP engaged with the CBs using community-based participatory research, resulting in 12 overarching categories of research priorities, including RH. Each category contains a general problem statement, as well as testable research questions stemming from the priorities identified by the CBs. It remains to be seen, though, if research has answered them. This study used these research questions as the basis for an adapted scoping study, querying extant literature on these research priorities.

**Results:**

These efforts resulted Our search found 132 peer-reviewed studies of RH since 1984, 111 of these pertaining to the CB’s research questions. These, however, were heavily weighted toward those providing fewer services and supervision (80%), and the research questions focused on RH operations (57%), though more recent efforts have investigated populations served (37%).

**Discussion:**

Though many RH studies fell within JEAP research questions, the literature has yet to reach an overarching consensus on best practices within each. Given the high degree of variation between types of RH programs and between geographic locations, such consensus may not be feasible or even desirable. Key elements of effective RH operations are discussed providing useful information for both researchers and practitioners to consider, as well as recommendations for future research.

## Introduction

Recovery housing (RH) aims to provide a supportive living environment for individuals in recovery from substance use issues. These settings are designed to foster community, accountability, and access to resources that promote long-term recovery, such as peer support (1). RH offers what could be a vital bridge towards reintegration into independent living and sustainable recovery. The benefits have been recognized by many US individuals and communities, who have aided in RH expansion through primarily grassroots efforts. Despite the critical role recovery housing may play, and due in part to the grassroots nature of the proliferation, a significant gap exists between this vital service and research.

Researchers often focus on rigorous data collection and evidence generation, emphasizing outcomes like abstinence and retention rates. In contrast, recovery housing providers and residents prioritize immediate needs such as housing stability, a sense of belonging, and mutual accountability. This disconnect can slow the examination and adoption of evidence-based practices, as well as funding mechanisms for services like RH, highlighting the need for greater alignment between the priorities of research and the lived realities of recovery housing stakeholders.

The Initiative for Justice and Emerging Adult Populations (JEAP; R24DA051950, see 2) was established to advance research on recovery support services tailored for emerging adults or individuals with criminal legal system involvement (and are of any age). This Initiative focuses on bridging the research-to-practice gap by building the research infrastructure for understanding and enhancing peer recovery support services and recovery housing to meet the unique needs of these two groups. In addition to several other activities toward these aims, JEAP co-created a set of research priorities pertaining to recovery support services with three community boards^1^ (CBs) using community-based participatory research (CBPR) (2). The current study extends these efforts by applying a scoping study methodology to ask whether research has addressed these priorities regarding RH.

## Recovery housing

Recovery housing is an umbrella term used to describe long-term recovery-oriented living environments that provide housing stability for those recovering from substance use issues (1). RHs can offer a variety of services but at minimum offer peer-to-peer recovery support and a safe environment free from drug or alcohol use. A key aspect of recovery housing is the incorporation of the peer-led social model of recovery, which all recovery residences have in common (3). However, the practical implementation of this concept varies widely and has necessitated the development of categorization to differentiate housing types.

Academic theoretical bases for RH stem from a few sources. While a full description of recovery science theory is beyond the scope of the current study, the relevant perspectives cite items like social structure and positive recovery supports as key to recovery and provided by RH (4, 5). From a practical perspective, this service inhabits an influential position within the Recovery Ecosystem (6) along the continuum of care (7).

The National Alliance of Recovery Residences (NARR) has developed four overlapping categories of increasing care intensity for this purpose and can be summarized as follows (8). Level I (“Peer-Run”) indicate peer-run RH, such as the Oxford House model, which include no external management, and the home is operated by the residents themselves. Level II (“Monitored”) RHs include more structure. External management provides support, enforces rules, and maintains structure. Level III (Supervised) houses supervise and monitor tenants to a greater extent. This level of housing typically entails a staff member available for 24/7 support as well as staff dedicated to counseling or directing group activities. Level IV (“Clinical”), sometimes referred to as Therapeutic Communities, tend to be a more institutionalized environment, with licensed staff members able to provide direct care. It should be noted, and is discussed below under Limitations, that this study focuses on Levels I-III due to the differences in both conception and in practice to Level IV housing, as well as due to guidance from the JEAP CBs on what is most needed as a focus of research.

A 2014 assessment of the evidence base for recovery housing by Sharon Reif and colleagues found the level of evidence for recovery housing to be moderate (1). They cited consistent positive outcomes, but limitations in study designs prevented a more favorable rating. Further, the Substance Abuse and Mental Health Services Administration included recovery housing in their Evidence-Based Practices Resource Center and created a document highlighting best practices and suggested guidelines (9). Today, there is estimated to be over 10,000 recovery residences across all 50 states and Washington, D.C., demonstrating a significant portion of the total recovery infrastructure (10).

## Study aim and rationale

This study sought to expand the field’s knowledge base for recovery housing by combining two methodologies: CBPR and a scoping study approach. Research in isolation does little good, especially when that research involves crucial topics like recovery support resources. Though the US has, thankfully, seen recent declines in fatal drug overdose, we continue to lose over 100,000 citizens every year (11). According to the 2022 National Survey on Drug Use and Health, nearly 49 million people aged 12 and over suffer from SUD (12). With the recognition that recovery support services serve an important role addressing substance use issues, particularly concerning long-term health, the field has seen a rapid increase in recovery science. The JEAP Initiative helped to lead the charge in this expansion, developing the set of Research Priorities described below, among other activities. Having established these priorities, the next logical step is to evaluate whether the literature has answered these questions. If not, then the field has a clear set of priorities to investigate. However, if these questions have been answered, then a translation gap likely exists preventing findings from reaching the people who could use it most. Therefore, this study used a scoping study methodology to query extant literature on research priorities expressed by people with direct experience and those who provide services.

## Materials and methods

This study combined the scoping study methodology recommended by Hillary Arksey and Lisa O’Malley (13) expanded by Danielle Levac and colleagues (14) with the CBPR work performed by the JEAP Initiative. The goal was to explore whether the peer-reviewed literature might provide answers to questions developed by the JEAP CBs through CBPR methods. The study, and this section (following a brief description of the methods used to develop JEAP Research Priorities), proceeded using the recommended categories, with Stages 1 and 4 adapted to incorporate the CBPR work, which included several questions being pursued rather than a single or a few questions as may be typical in a scoping review.

### Establishing on-the-ground research priorities

Beginning in 2020, the JEAP Initiative established three CBs consisting of young adults (age 18–25) in recovery from substance use issues, people with criminal legal system involvement and in recovery, and payers and providers of recovery support services (15). Though some turnover did occur over the proceeding years, CB membership^2^ throughout this period ranged between 8 and 10. Relevant to the current study, all CB members were familiar with RH programs and their importance, and most engaged with the service in the past. As examples, on the Payer & Provider board, one member helped create the National Alliance for Recovery Residences, and two led or worked in organizations that feature this service for people released from incarceration. A member of the Young Adult CB facilitated a collegiate recovery program, which included housing services, and a Justice-Involved member worked on statewide efforts for recovery housing. Many others had utilized recovery housing in their recovery journey, so there was direct and peripheral experience throughout the CBs.

While these CBs serve many functions within the JEAP Initiative (e.g., advising on hiring of postdoctoral fellows, selection of trainees, and development of research projects), a primary function was to develop a set of research priorities. Utilizing community-based participatory research (CBPR) methods (2), this process began with brainstorming across several CB meetings, resulting in a large list of potential items, with each CB devoting 45 min to the exercise. In most cases, a JEAP investigator was present, but the discussions were facilitated by cofacilitators elected by each Community Board. After synthesizing the CB comments from the brainstorming meetings into shorter statements, the JEAP team sent each member a list of their statements, ensuring their accuracy. CB members had an opportunity to add to the list or clarify statements in a follow-up 30-min meeting. Then, each member was given the full list of ideas generated by their Community Board and asked to rank them ideas into “High,” “Medium,” or “Low” priority categories using Microsoft Excel and UXtweak card sorting platforms (16). The JEAP research team categorized those items ranked as high and medium priority into general categories, resulting in 12 overarching themes, one of which became the recovery housing category used in this project. The team also converted the statements presented as priorities in previous steps into testable research questions, with the ultimate goal of inspiring researchers to pursue answering these questions.

The research team and all three CBs then met for a two-hour “Community Board Retreat” to complete member-checking (a verification technique to ensure the validity of findings, particularly relevant in CBPR where collaboration and mutual understanding between researchers and community members are crucial) (17, 18), during which CB members provided guidance on refining the research priority categories and research questions within each. CB guidance was then incorporated, resulting in the Research Priorities available on the JEAP website (19). Table 1 provides the RH Problem Statement and Research Questions, as well as the broad category for each Research Question used in this study and which CB each originated from; some questions were raised by more than one CB. These are presented in the order they appear in original, and are numbered thusly, so some broad categories are included out of order.

**Table 1:** Recovery housing problem statement and research questions prioritized by community boards (CBs) through community-based participatory research methods.

	*Problem Statement: The elements of recovery housing that make it most effective are under-researched – including internal operations, accessibility, connection with other services, and environment.*		
Research question number	Broad category	Research question	Community board
1	Operations	What are the key ingredients of recovery housing (e.g., accountability, social support)?	J
2	Operations	What aspects of recovery housing, and the combination of services within them, provide the most help?	J, P
3	Economic impacts	Beyond recidivism, what are the economic impacts of these? For example, do they reduce the use of healthcare and Medicaid dollars, child welfare system costs, etc.?	P
4	Populations	What populations are served by different types of recovery housing and how do outcomes differ across housing types and population groups?	P
5	Populations	How are different populations accessing and paying for recovery housing?	P
6	Populations	What strategies can increase representation of people of color within recovery housing? How can recovery housing be more welcoming for people of color?	Y
7	Populations	What drives the lack of recovery housing specifically for women, especially housing run by women?	Y
8	MOUD	What prevents MOUD/MAT from being accepted as legitimate recovery in different contexts: in recovery housing; in the justice system (e.g., treatment courts, law enforcement, prison staff); in treatment providers; in social networks?	J, P
9	MOUD	What policies and strategies have led to increased acceptance of MOUD/MAT in different contexts, and how can that be replicated?	J, P
10	Peer Recovery Support Services	How can peer support specialists effectively assist individuals with the shift from transitional housing to long-term housing, especially given the housing crisis that is felt more broadly?	J
11	Peer Recovery Support Services	How can recovery support services, like housing, coordinate with jails and prisons to create long-term treatment plans, seamless supports, and continuity of care for those returning to the community?	J
12	Populations	Are emerging adults being incarcerated just because there aren’t supports for them (e.g., no foster parents, nowhere to go)? Are there recovery housing options for them?	J

J = Justice-Involved Community Board (CB), Y = Young Adult CB, P = Payor & Provider CB.

### Stage 1: identifying research questions

Our primary deviation from the standards set in Arksey & O’Malley (13) and Levac et al. (14) is the incorporation of CBPR in the development of the research questions and the organization of the results. As described above, the JEAP CBs provided a set of 12 research questions. For a scoping review, this presents an issue to have such a large number of research questions. The study team took two measures to lessen the workload associated with the large number of questions. First, the study team distilled the 12 original Research Questions into five broad categories by unanimous consensus over two separate meetings and via email communications (see Table 1): Operations (Research Questions 1–2), Populations (Research Questions 4–7 and 12), Medications for Opioid Use Disorder (MOUD; Research Questions 8–9), Peer Recovery Support Services (Research Questions 10–11), and Economic Impact (Research Question 3). The second measure involves an additional categorization step, described below.

### Stage 2: identifying relevant studies

With guidance from a specialized university librarian, we developed a comprehensive search strategy. The databases searched included Ovid MEDLINE, PsychInfo, Sociological Abstracts, and Web of Science. The search consisted of both controlled vocabulary (e.g., MeSH terms, subject headings) and free-text keywords and was structured around two main concepts: (1) recovery homes and related facilities and (2) substance use. For the former, we included a broad range of terms, including terms often outside the scope of recovery housing for sake of completeness. These included terms such as group homes, halfway houses, sober houses, therapeutic communities, as well as terms more closely associated with recovery homes such as recovery residences and Oxford houses. For the second concept, we included a similarly broad depiction of substance use with terms including but not limited to drug abuse, substance addiction, chemical dependence, and drug habituation.

To ensure completeness, Boolean operators (AND, OR) and proximity operators (e.g., NEAR, ADJ) were employed to expand the search results. That is, these terms were used individually, as well as in combination with and proximate to the others. Each search was tailored to each database’s syntax and thesaurus. For instance, in Web of Science, the ALL and TS fields were used, while PsychINFO employed DE for descriptor terms. Ovid’s search included the use of exploded MeSH terms and field-specific searches (ab, kf, kw, ti). Sociological Abstracts utilized NOFT (non-full text) searches and MAINSUBJECT.EXACT.EXPLODE for more comprehensive concept inclusion. A full list of search terms and logical functions used for each literature source can be found in the Appendix.

### Stage 3: study selection

**Figure 1:**
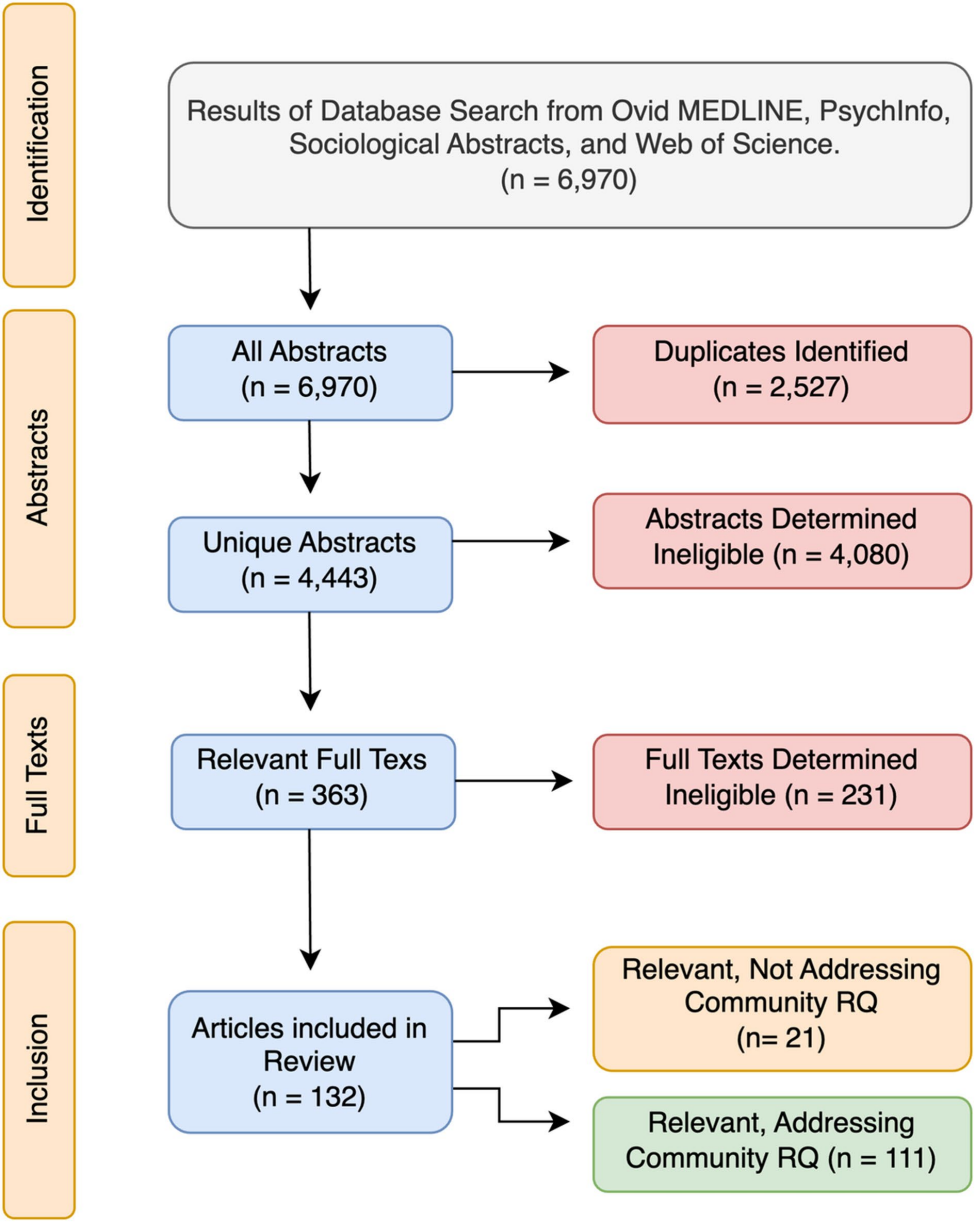
A visual representation of Stage 3: *Study Selection.*

Figure 1 summarizes the steps in this stage. Between the four databases, our initial search yielded 6,970 articles. Removing 2,527 duplicates left 4,443 abstracts to be reviewed. Following the guidance of Levac et al. (14) we completed study selection as an iterative process involving two distinction stages of article selection where the content of the articles themselves helped inform selection. Also following best practices, the research team met at the beginning, multiple midpoints, and the end of the abstract review section to discuss uncertainties and to refine the selection criteria (14).

Per unanimous decision by the study team, and in consultation with the JEAP CBs, we limited the selection to studies written in English, with populations within the US and Canada, and published in peer-reviewed journals (i.e., not “grey literature” like dissertations or reports). Our study period went from January 1984 to October 2023. This decision was made considering both the scarcity of articles and the lack of similarity to current RH practices for anything prior to 1984. The research team also decided to exclude studies centering on the effects of Covid-19 and RHs unless the study added significant information to the field which could be generalized outside of pandemics. Studies using RH populations to investigate non-RH topics (e.g., develop psychometric measures, psychological principles) were also designated as exclusions. For instance, the 2023 study by Ted J. Bobak and colleagues (20) studied whether psychiatric comorbidity (with a substance use issue) impacted Oxford House residents’ social capital. Though this study took place within a RH, it did not investigate elements generally relevant to RH or the JEAP RQs. Finally, Level 4 RHs, or “Therapeutic Communities,” were excluded from the study due to the relative conceptual distance from the other three other levels; additionally, there is a wealth of articles on Level 4 RHs so that subfield is well-established, and the CBs (see below) wanted to spotlight the need for research on the less-established levels of RH.

We met with all three community boards to confirm that the inclusion and exclusion criteria were appropriate for this study’s goals. We were specifically interested in hearing their views on the exclusion of Level IV RHs. The CBs largely agreed with the exclusion of this category, with a member of the Young Adult CB stating, “It makes sense to keep Level IV under the treatment umbrella, but in my mind, it’s separate.”

Two authors (PH, CT) reviewed 4,443 unique abstracts where 4,080 were excluded leaving 363 full texts for further consideration. Further review during the proceeding stages excluded 231 (described below), leaving 132 studies for the final stage.

### Stage 4: charting the data

We used the categories of the JEAP CB Research Questions described above to chart the study data. Practically, this involved two rounds of full-text review conducted with the MAXQDA qualitative analysis software (21). First, studies were categorized by research question broad category/categories addressed within them. Any findings related to a research question were included. In total, 21 studies did not address a RQ but met all other inclusion criteria, which were preserved as “relevant to examining RH but not JEAP RQs,” meaning the final number of studies considered is 111. This round also included coding for information like study location and time period. NARR level was determined not only from explicit statements within studies but also from descriptions of the RH under investigation. To expedite compiling information from studies and create a preliminary master table two authors (CT, JT) placed study contents into a Large Language Model (LLM), GPT-4o-2024-05-13 (22), where it returned a brief synopsis of each study and categorized its research design. This summary and research design designation was subsequently verified and often edited by the authors to ensure accuracy.

In the second round, authors divided the studies into areas of focus (i.e., two researchers were responsible for the Operations section, two researchers for Populations, etc.). In this portion, authors confirmed the categorization from the first round and differentiated studies where a study simply acknowledged a RQ from studies in which a RQ was the focus. During this process, authors also verified the LLM generated summaries, ensuring accuracy of the synopsis and study design.

Through both rounds, studies were additionally excluded that did not meet the criteria described above. Though the abstract review filtered out most non-RH studies, and initial coding a better idea of which studies pertained directly to the JEAP Research Questions, a full text review was required in many cases (231 to be precise) to determine whether a study dealt specifically with RH (e.g., not simply a study performed within the RH context) and fell within the 12 Research Questions.

### Stage 5: collating, summarizing, and reporting the results

During the second round of coding described above, individuals on the study team responsible for each category further coded studies regarding each study’s main research question, main results, limitations, and future directions. This final coding stage provided material for the results presented below.

## Results

Because this scoping review includes numerous research questions, the results are presented as an overall summary of what was represented in the 111 studies and then organized into the distilled categories of the JEAP CBs’ RQs: Operations, Populations, MOUD, Peer Recovery Support Services, and Economic Impact. In the section on the JEAP CBs’ RQs, where the number of studies warranted it, descriptions of studies are summarized by NARR level and/or by the specific RQ.

### Overall characteristics of included studies

#### Research question category and level of care

The Appendix provides a full table listing all the publications retained for final review (as well as RH-focused studies that were not relevant to the RQs), including title, author (s), year, location, NARR level, CB RQs addressed, RQ that was the primary focus, a brief synopsis, and study design category. Table 2 shows distributions of the studies within the broad RQ categories and per NARR level. As this information displays, studies on RH overwhelmingly focus on general operations and populations served. Additionally, the distribution of NARR levels studied weighs heavily toward Level 1, primarily labeled Oxford Houses. One study did not present sufficient information to determine what NARR level was under investigation and 15 covered more than one.

**Table 2:** Number of studies representing each research question category and NARR level.

RQ categories	Number	NARR Level	Number
Operations	66	1	63
Populations	38	2	29
MOUD	5	3	6
PRSS	1	Multiple	15
Economic	1	Unknown	1
Total	111	Total	111

NARR, National Association of Recovery Residences.

#### Year, location, and study design

**Figure 2:**
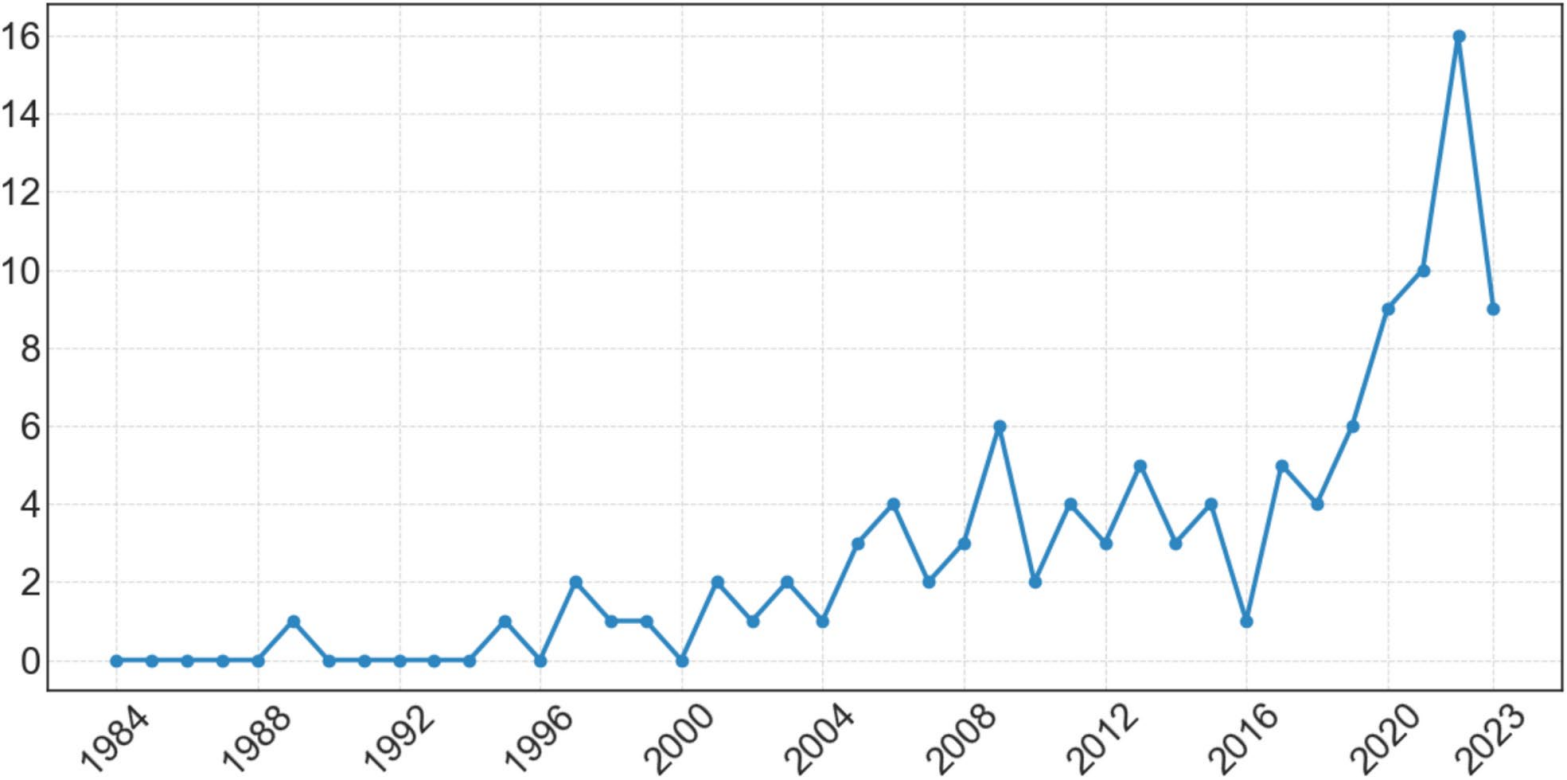
Number of community relevant RH studies per year.

Figure 2 displays the number of RH studies per year addressing a question from the community board, with years represented on the x-axis and number of studies on the y-axis. Note that our initial search returned studies prior to the first listed here, from 1989, but those were excluded during the review process for not meeting other inclusion criteria. These studies increased year-on-year, peaking in 2022 with 21 studies. Note also that this scoping study’s period ended October 2023, so 2023 is likely underrepresented.

Determining the study period and location of most included studies was exceedingly difficult. Many studies did not mention the time period of their project or even the location. And, though some worked with data from previous efforts, few directly labeled their data as such.

Table 3 provides information on the types of study design used. Altogether, the emphasis is largely on quantitative methods, though only three of these were randomized-controlled trials. Qualitative designs were used in 21 studies, and four used mixed methods.

**Table 3:** Study design distribution.

Study design category	Count
Quantitative	86
Randomized controlled trial	3
Non-randomized	83
Qualitative	21
General qual.	7
Interview	7
Focus group	5
Ethnography	1
Case report	1
Mixed methods	4

#### Demographics studied

Given the large proportion of RQs pertaining to the populations served, the study team also attempted to compile demographic information (sample size, age, gender, and race/ethnicity) for the articles reviewed. Though this information would not amount to an empirical study of the populations served by RH, it would provide a view of those served within the current sample of studies. Unfortunately, the inconsistency in reporting made analysis more cumbersome than appropriate for the current study.

The Appendix provides all details that could be gleaned from included studies. Complete reporting was considered if both the number of participants and proportion of the sample for each category was listed (i.e., “10 male participants, 50% of the sample”), or if this was easily inferred from given data (e.g., “10 participants, with eight male and two female”). We also assessed if age was reported as a mean and standard deviation per reporting recommendations for a continuous variable (23, 24). Of the 111 studies included in analysis, 42 reported completely, 57 partially, 10 not at all, and two reported in other places (i.e., samples from a previous study). Some of the studies noted as partially reporting indicated things like the number or percentage of one or two racial categories without indicating what the rest of the sample represented. It should also be noted we marked some of the studies as reporting completely, though they reported partial information, due to the nature of the study. For instance, Graham et al. (25) investigated the impact average age per Oxford House would have on average house income. Another point to consider is that studies that reported partially range quite a bit in missing data. Some studies neglected to include smaller items like the standard deviation of age, while others reported but a few descriptive statistics.

### JEAP research questions

#### Operations

Since the two RQs comprising the Operations category represent such similar sentiments (see Table 1), consideration of these two has been folded together. As with other categories, studies pertaining to RH operations tended to be in NARR levels 1 and 2. Altogether, studies within this category represent a large range of elements with RHs that might be considered “key ingredients” (as stated in RQ1), relevant to the type of housing under investigation. As an example, one study evaluated the importance of a house manager’s role (26), and another the importance of training RH staff (27). Such issues would not apply to analyses of Oxford Houses, which have no staff or managers.

While RQs 1 and 2 alluded to what might be considered internal operations questions – “key ingredients,” and “aspects of recovery housing … [which] provide the most help,” – a pattern quickly emerged from included studies pertaining to the scope under investigation: internal elements (e.g., capacity, operating principles, social supports), external (e.g., neighborhood characteristics, affiliation with other services, social stigma), or some combination. Of the research questions asked in these studies, 50 pertained to internal operations, 12 external, and 18 some combination (note that some studies asked multiple research questions, so the total amount here sums to more than the number of studies within the category).

Within the findings from these studies, several notable themes emerged that provide results directly relevant to these RQs. Namely, support systems and social dynamics, community and stakeholder relations, and the interplay between internal operations and community context. Of findings under review, 33 either directly studied or attributed positive results to this idea of support networks (e.g., 28–31). Within this category, 18 studies dealt with the social dynamics of RH residents, indicating that the integration into RH showed benefits for both new entrants and more senior residents (e.g., 4, 32). That is, ensuring new residents become integrated into a RH benefited these new residents by creating a supportive structure around them, *and* those who performed this integrative function (more seasoned residents) benefited by being a part of this supportive structure. Additionally, the view that providing a support system is an important element of RH was reflected by people at state agencies (31), and even property owners who rent to RH programs (33). Study of specific programming elements in RHs was rare, though a few exceptions exist. For instance, one study looked at the inclusion of occupational training, finding an association with direct outcomes like employment, but also with indirect items like self-esteem and quality of life (34), indicating the way in which elements of recovery dynamically interact.

Many papers also studied external factors, under the premise that, “… it is insufficient to study the effectiveness of community-based services without examining characteristics of the community context in which those services are delivered” [(35), p. 107]. Some of the elements studied include the types of neighborhoods RH inhabit (e.g., poverty levels, property values) and features within them (e.g., liquor outlets, mutual-aid meetings) (e.g., 27, 36). Though some findings indicated these factors influence outcomes (e.g., access to public transportation; higher income neighborhoods), the primary theme was that RH, and residents, were resilient—at times even finding that “negative” community characteristics like the presence of “temptations” offer opportunities for people to practice denial strategies (37). Many studies also looked at community relations, both as important to the continuing operation of RHs and as an element impacting resident outcomes. Altogether, themes emerged indicating best practices include engaging with the neighborhood and broader community, that a “good neighbor” policy reduces stigma and logistical challenges (35, 38). Finally, the literature has expressed the need for policy adjustments like increases in funding, reductions in regulatory barriers, and integrating RH more deeply into formal care systems toward sustainability and effectiveness (26, 27, 39, 40).

Neither internal nor external elements occur in a vacuum. Internal social dynamics are bound to be influenced by context. Mericle et al. (39) provides a good example of both internal and external, and perhaps the closest study of “key ingredients” to-date, particularly regarding Level 2 RHs. Their study looked at a host of RH operational and principled characteristics, including internal items like adherence to social model and 12-step principles, level of fees and whether meals were provided, resident characteristics (single gender programs vs. mixed), as well as external factors such as neighborhood characteristics, grouping of RHs (i.e., organizations with a number of RHs), and RH affiliation with other services. Though we do not have space to discuss all this study offers, their work found several elements associated with positive outcomes (e.g., abstinence, employment). RHs that were part of a larger organization, affiliated with treatment services, charged higher rates (>$600 per month), and housed men showed an association with more positive outcomes. These results, however, decreased in significance when the variables were put together in a multivariate multilevel model.

#### Populations

Unlike the Operations category above, the RQs contained in the Populations category represent distinct concepts. Therefore, they are covered here separately. As Table 4 displays, these studies were weighted toward RQ4, concerning how different populations are served by RH.

**Table 4:** Distribution of studies within populations research questions.

RQ Number	Question	Number of Studies
4	What populations are served by different types of recovery housing and how do outcomes differ across housing types and population groups?	14
5	How are different populations accessing and paying for recovery housing?	0
6	What strategies can increase representation of people of color within recovery housing? How can recovery housing be more welcoming for people of color?	10
7	What drives the lack of recovery housing specifically for women, especially housing run by women?	11
12	Are emerging adults being incarcerated just because there aren’t supports for them (e.g., no foster parents, nowhere to go)? Are there recovery housing options for them?	3

##### Populations served by housing type and outcomes (RQ4)

This section provides a discussion of the articles represented between NARR levels 1 and 2, and those without a level-of-care designation.

##### Level 1

Most studies investigating population question RQ4 that focused on NARR Level 1 (e.g., Oxford Houses) examined psychiatric severity among its participants. Article research questions examined the prevalence of psychiatric comorbidity among men and women (41), psychiatric severity over time (42), and types of psychosocial distress (43). Another study focused on assessing outcomes among residents with physical health disabilities (44). There were also several studies that examined People of Color and women in the Oxford House environment that will be discussed in the RQ6 and RQ7 sections below.

Majer and colleagues (45) demonstrated that Oxford Houses serve as effective supportive recovery environments, facilitating residents in managing psychiatric severity that could impede recovery progress. However, their findings indicated that psychiatric severity exerted a more pronounced influence on individuals engaged in medication-assisted treatment (MAT). Notably, the impact of psychiatric severity on stress was attenuated among MAT participants who were housed with at least one other resident also utilizing MAT. This suggests that peer support within MAT context may mitigate some of the adverse effects of psychiatric severity on recovery outcomes.

In their study comparing CJS-involved men and women living in Oxford Houses, Coleman and fellow researchers (43) found the length of residency had an inverse relationship with psychosocial distress and that women experienced a higher level of distress compared to men. They also found that while women experienced more mental health and SUD symptoms compared to men, women who resided for more than 2 years had a lower psychosocial distress score than men living in the Oxford House the same length of time.

Only one study focused on physical health disabilities for those residing in Oxford Homes (44). Specifically, Alverez and colleagues assessed differences between Oxford House residents who were deaf and those who could hear. The study findings demonstrated that there were no significant differences between deaf and hearing residents in months of sobriety, sense of community, and abstinence self-efficacy.

##### Level II or III

With the exception of articles that included age-related outcomes discussed in the RQ12 section, only one study within the RQ4 populations category included residents living in NARR level II and III homes. Mericle and colleagues (46) used qualitative interviews to learn about the experiences of men who have sex with men living in a recovery residence specifically serving this population. The authors found that LGBTQ-specific residences effectively address common barriers such as housing and finances but also provides additional support to address sexual minority stress, such as discrimination, stigma, and internalized homonegativity.

##### Programs

Several articles did not specify a NARR designation. Like articles within the Oxford House category, some studies investigated psychiatric severity among residents. Results show that residents with high psychiatric severity had higher alcohol and drug severity on ASI scales (47), but that living in SLH environments was associated with lower drug severity, and improvements in both psychiatric severity and housing status (48). Other studies compared resident characteristics prior to and following completion of recovery home programs. These studies found residents faced several deficits undermining effort to initiate and sustain recovery with many experiencing homelessness, unemployment, depression and anxiety (49, 50). Researchers found despite these deficits, many of the most vulnerable people completed the program (49), and that programs improved rates of substance use, criminal justice involvement, employment (50), with a small number of people reporting return to use (49).

The final study compared differences between employed and unemployed men living in SLH and sought to understand how men within SLH may stigmatize and discriminate against each other based on employment status (51).

##### Accessing and paying for recovery housing (RQ5)

Although no studies looked at how different populations access and pay for RH, one study did look at whether an association exists between demographic variables (level of education, race/ethnicity, age; the sample was 100% male) and employment for Oxford House residents, finding none (51). This study did, however, find a tie between unemployment and those who felt discriminated against due to recovery status. The authors looked at a cross-section of data, so they could not determine the direction of this relationship—whether unemployment influenced the feeling of discrimination, or vice-versa—nor make causal claims.

##### Strategies to represent people of color (RQ6)

Several articles focus on People of Color, often underrepresented in recovery housing, including Latines, Indigenous and Native people, and African Americans. Specifically, six articles focused on Latines, two articles focused on recovery housing for Indigenous and Native people, one article focused on exploring differences in characteristics among African American men and women, and two articles focused on assessing recovery outcomes or employment between African Americans and other racial/ethnic groups.

Of the six articles focused on Latinos living in recovery housing, three focused on Latine residents’ experiences living in traditional versus culturally modified Oxford House recovery homes. While one article described Latine residents’ differing thoughts on the need for a Latine or Spanish-speaking Oxford House (52), others found living in culturally modified homes associated with better employment outcomes (53) and improved collectivism that may have contributed to reduced time spent in the home with less relapse (54). The remaining two articles focused specifically on Latina women. One examined homophily among residents and counselors and found that connections were important to help Latina women increase involvement in recovery housing and transform their lives (55). The remaining article found that women in the culturally specific recovery home remained in treatment longer and were more likely to have a satisfactory discharge from their recovery home (56).

Two papers examined Oxford Houses for Native people. These studies investigated whether the Oxford House model could be successfully adapted for the Suquamish Tribal reservation. Findings suggest that the recovery home values, particularly the communal and democratic way in which Oxford House recovery homes operate, are compatible with the values of Native Americans (57) and promote friendship, trust, and mentorship, factors critical to recovery (58).

Belyaev-Glantsman and colleagues (59) explored differences in residents’ employment and income and found higher employment rates among African Americans compared to European Americans, though no significant differences in income between the two groups highlighting potential pay disparities. The final article examined racial and ethnic differences in changes in a multi-factor recovery item, finding rates of improvement were higher among participants who identified as Black compared to other racial/ethnic groups (60).

##### Lack of recovery housing for women (RQ7)

Several articles focused on varying topics related to women in the recovery housing setting, some of which include, outcomes of a trauma-informed, gender-responsive recovery home, leadership roles among female residents, the impact children living in Oxford houses have on sense of community, and women with eating disorders.

Five articles examined women newly admitted to the SEEDS program, a trauma-informed, gender-responsive SLH. Studies show that while women entering the SEEDs program reported substance use and DSV victimization and enter homes with diverse needs, participation was associated with reductions in alcohol and drug use, DSV, financial and housing insecurity (61). Another study examining this program found that participants with increased financial worries and housing instability had lower perceptions that the SLH was trauma-informed, pointing to the need for more trauma-informed practices for women higher in financial worries and facing housing instability (62). Another study on the SEEDs program found that younger women were less likely to stay in the program for 3 months or more compared to older individuals. Further, women with higher levels of financial worries were more likely to stay more than 3 months in the program (63). The final article explored the relationship between sense of community (SOC) and mental health symptoms, with the majority of women reporting high levels of SOC which was related to lower severity of mental health symptoms. However, despite the link between SOC and mental health levels, the authors call for more research to explore the sequencing of this relationship (64).

In regard to women in leadership, Davis and colleagues (65) found that women thrive in all-female, communal leadership environments, which foster strong bonds and improve home operations. Olson and colleagues (66) similarly highlighted that communal settings in Oxford Houses can empower women with past trauma, low-self-worth, and unhealth relationships. Timpo and colleagues (67) further emphasized that women in leadership roles within recovery homes may boost their self-esteem, crucial for those with a history of SUD.

Two articles focused on women with eating disorders (EDs). Studies found that 39% of the sample met criteria for an ED and that longer stays in an Oxford House was associated with higher scores for body image efficacy (68). Women with substance-related disorder and ED can benefit by living with other women in a communal style living environment offered by Oxford Houses, however women with eating problems perceived an inability to forge harmonious relationships with other women in the home, calling for more research into how feelings of disharmony can undermine the recovery process (69).

Other articles focused on the influence of different factors, such as social support, attitudes toward children living in recovery housing, and perceived economic autonomy on recovery housing outcomes among women. Specifically, one study found that the presence of Oxford House members in personal social networks significantly predicted retention for women living in recovery homes, while reciprocal responsibility was linked to the number of paid workdays for women (70). d’Arlach and colleagues found that positive attitudes toward children living in recovery housing were strong predictors of a sense of community and suggested that children positively impact both mothers and women without children (71). The last article that focused on women examined the relationship between perceived economic self-sufficiency, social support, and substance use among participants following their stay in a long-term treatment program and found that reductions in substance use was associated with higher economic autonomy (72).

##### Recovery housing options for emerging adults (RQ12)

Three studies investigated elements relevant to this category, though none directly addressed the issue of recovery housing options for emerging adults. Krentzman and colleagues (73) compared differences in baseline characteristics between emerging adult women and women 30 years and older and found emerging adult women were more likely to be asked to leave their sober living home for breaking rules. Researchers suggest sober living homes may need to consider different types of programs and policies that account for the differences between younger and older women.

Two articles focused on age differences more generally and associated outcomes among residents. One study tested if individual and group characteristics could predict length of stay and found that older age and older age of fellow residents were the best predictors of continued residence (74). Another study examined the relationship between age and income to overall household psychological sense of community and found that houses with larger age and income heterogeneity were associated with a higher sense of harmony (25).

### Medications for opioid use disorder

This scoping study revealed five studies addressing the issue of medications for opioid use disorder (MOUD). Though the JEAP CBs’ questions pertained to the stigma around MOUD, the discussion here includes those that looked at MOUD from any perspective. The first item to note is that one study evaluated the mediating impact of social support on the relationship between stress and recovery outcomes, and whether this relationship was moderated by use of MOUD (45). Results indicated that social support seemed to reduce the impact stress has on recovery and, importantly, that MOUD had no significant moderating effect.

Two studies examined resident participants’ attitudes about MOUD in recovery residences. Majer et al. (75) conducted a study of 90 participants in Oxford Houses in Maryland, finding mixed results.^3^ Those not on MOUD indicated negative views and those using MOUD showed mixed feelings, particularly between different types of medications (methadone showing the largest proportions of negative associations). This study was expanded a few years later with similar, but less severe, results in negative attitudes surrounding MOUD. Each of these studies found that negative stigma associated with MOUD may even be found within the recovery community itself.

To address the negative stigma within the recovery community, Bobak et al. (76) created an educational pilot program, with promising results indicating MOUD education and awareness may be a significant predictor for whether MOUD is accepted and may also influence negative stigma held within the community.

Another study examined the extent of MOUD acceptance in RH managers and staff, finding 98.4% of RHs permitted at least one form of MOUD, but only one allowed all three (77). When comparing the acceptance rates across medications, researchers found that buprenorphine was much more likely to be accepted than methadone. This difference was more pronounced in residences where tapering was encouraged. House managers who encouraged tapering were significantly less accepting of methadone.

### Peer recovery support services

This review only found one study that investigated peer recovery support services (PRSS). Mericle et al. (78) examined a group of Level 3 RHs in Texas which incorporated the services of recovery coaches (a synonym for PRSS) into residents’ care, among other elements. While this work does not offer empirical analysis of PRSS in the RH context, it does provide analysis of the adherence to the social model philosophy within this RH setting. Authors found that “social model principles dominate” (p. 357), but did not reach the level of full adherence, discussing the possibility of a move away from these principles due to evolving concepts of recovery (e.g., harm reduction vs. abstinence) and the professionalization of the recovery field.

It should be noted that a significant portion of the literature on RH inherently incorporates some aspect of peer support, if not PRSS in name, due to the basic design of recovery housing, especially Level 1 programs. Oxford Houses, by definition, include peer support, being peer-led, democratically run support programs prioritizing substance use recovery by creating a supportive community of peers. The bulk of literature on these programs illustrates support among peers provides key elements that lead to positive outcomes. Finally, as mentioned in the one study within this category, other RH programs may not provide services like PRSS directly, yet residents can, and often do, access them.

### Economic impacts

Only one study directly investigated the community-focused economic impacts the JEAP CBs indicated (e.g., healthcare or other services costs) relative to RH participation. Koroloff and Anderson (79) looked at an alcohol-free living center serving people experiencing homelessness in Portland, OR. Their work found substantial improvements in employment and reductions in use of other services (e.g., short-term detoxification). Additionally, 15 of those included under other RQ categories did look at employment and income characteristics of RH residents, overwhelmingly finding improvements based on participation. Another study found Illinois Oxford Houses tend to be located in areas with higher areas of unemployment, recommending that future development consider this and aim for areas with better employment prospects (36).

## Discussion

This project sought to combine CBPR and scoping study methodologies within the topic of recovery housing. We followed the guidance of recommended scoping study approaches with a few modifications that allow the incorporation of CBPR. First, research questions were determined by CBPR with the JEAP Initiative’s three Community Boards as described above, rather than the more traditional, researcher-led route. This design was preferred for two primary reasons. First, RH as a construct does not fit neatly into empirical categories. Though valiant efforts from organizations like NARR have begun the process of formalizing RH into consistent categories, the service diffused across the US haphazardly. The basic definition of RH continues to differ across the country and has differed quite a bit over time. The second motivation for incorporating CBPR has to do with the impact and practice of RH. The research field, policymakers, and the public generally look to content experts (i.e., academics) as a knowledge resource, yet many of these experts lack direct experience with either providing or receiving the service they research with having lived experience where access to the service could have been helpful, and/or being embedded in a community in direct need of the service. More recently, the idea of “context expertise” or “experiential expertise” has gained traction in research and policy arenas. That is, knowledge from the people who provide and receive services under investigation. This phenomenon has become particularly impactful in the study of treatment and recovery support services (80, 81).

While the research questions used in this scoping study reflect those developed by JEAP CBs, the primary goal of this study can be boiled down to one: Does the literature answer the questions about RH people on-the-ground want answered? The results of the current study indicate that most of these remain unanswered. Within the categories of research questions used above, the richest body of evidence explores RH operations, though this literature has not settled on a universal set of best practices. The need for investigating how RH serves different populations (or not) is reflected in both JEAP CB RQs and a marked increase in this line of inquiry, but specific direction regarding these issues has yet to emerge. Study of issues like MOUD, PRSS, and broad economic impacts of RH have seen some activity recently, though these represent the beginnings of empirical investigation.

A note of clarity regarding the direction of research questions asked. The clear intention of JEAP CBs pertained to those elements that make RH successful, yet many included studies framed research questions as looking at the elements of RH that might influence outcomes. While the distinction may seem small, we want to be precise. The ultimate goal is, of course, to improve the lives of people with substance use issues. Achieving such positive outcomes, however, occurs along a complex path within a complex milieu. Such complexity requires precision in conceptualization.

It is also important to note that none of the JEAP RQs pertained to effectiveness. That is, the people who participated in JEAP CBs did not question whether RH services work. To be certain, the preponderance of evidence presented in RH literature *points toward* its effectiveness, *and* people with direct experience providing and utilizing RH speak highly of its benefits. That said, the basic question of “Does recovery housing work” still lingers, with room to increase the number and rigor of studies establishing effectiveness. Of the studies incorporating a quantitative methodology, only three were randomized-controlled trials. And of the remaining 83 quasi-experimental studies, 31 were cross-sectional, making causal inference and causal direction difficult. Given this, and the poor reporting we found (see section on Demographics above), additional studies examining the effectiveness of RH are warranted, particularly more rigorous work.

The state of this research suggests a need for more rigorous reporting practices, including demographic information, as well as specific study period and location. Future studies should strive for complete and transparent reporting of participant demographics, actively recruit diverse populations to improve representativeness, and acknowledge prior sample derivations to enhance the clarity and replicability of findings.

However, this is a nascent field of research and as more researchers are developed and more research funding is provided, the landscape will likely shift. In that process, we recommend that priorities of the community, such as the research questions identified by the CBs, drive the development of research to ensure the highest relevance and validity of results. The remainder of this section discusses each RQ category.

### Operations

“Key ingredients” derived from RH studies include support systems and social dynamics, community stakeholder relationships, and the interplay between internal and external factors. The notion of support systems permeates the breadth of recovery science literature and, in fact, provides a premise for services like peer recovery support services (4, 5). Within the RH literature, as well, many studies cite the social supports as not only critical to success within RH programs, but that RHs themselves cultivate such support, leading to improvements in many outcome domains. Therefore, the notion that “the opposite of addiction is connection” may provide solid guidance for RH programs (82). Emphasizing social integration of residents—whether in the peer-led, Oxford House sense, or some formal process in RH in higher levels of care—would provide the first key component. Further, the few studies that evaluated specific programming elements within RHs indicate that different elements of recovery interact dynamically and, relevant to the current discussion, RHs can manage these to positive results. For instance, Martin (34) looked at an occupational intervention, yet found addition impact on self-esteem and quality of life, possibly indicating a positive feedback loop. Thus, ensuring RH residents can gain educational or employment skills can add to self-esteem, which can then lead to stronger recovery, which continues to improve self-esteem, etc.

The literature also points toward intentional management of community relations, citing the need for a “good neighbor” policy [e.g., (35)]. More than simply avoiding negative dynamics (e.g., stigma toward RH and/or residents), some studies allude to RH residents gaining recovery capital by taking responsibility for these community relations—that the road to integration into society starts with such processes. Further studies advise consideration of specific factors that may influence resident outcomes like overall capacity, explicit connections to other treatment and recovery services, resident fees, and location. Though the literature does not point toward a specific set of items to look out for, it does rate such consideration as important.

Contents of the studies within this category illustrate a likely reason the RH literature has yet to reach consensus. Since this service diffused organically across the US—responsive to a variety of (primarily local) conditions like needs, resources, and policies—generalizability becomes difficult. The variation between types of recovery housing and location presents a counter to generalization. For instance, the individual accountability that comes with making democratic decisions in an Oxford House model (NARR Level 1) may improve residents’ self-efficacy (83), but such a feature would not be directly applicable to sober living houses (NARR Level 2). Variation in location can have a similar impact, with local conditions influencing the effectiveness of specific RH elements. In cases such as RH, though, perhaps generalization might not provide the most reasonable goal. After all, these services aim to help people in their community, which often requires local adaptation rather than direct replication. Such a need for variation, though, does not detract from the need for more, and more rigorous, study of RH.

Altogether, examination of the studies within this category point toward the need for more robust conceptualizations. Though a universal set of proscribed best practices might not be advisable, or even possible, in the RH context, research and practice would benefit from a framework that describes the potential units-of-analysis, possible key ingredients within each, and ways in which these may interact to create outcomes. Application of contemporary theoretical work like Recovery-Ready Ecosystems may provide a guide (6).

Finally, the general lack of rigor in these studies presents difficulty in determining a set of best practices. For instance, a paper investigating RH characteristics found an association between programs with more capacity and lower employment outcomes (39). It could not be determined whether the number of people in the house had some effect on employment, or if these programs simply featured less financial burden for residents, lowering the need for employment.

### Populations

Overall, there is a lack of research that addresses strategies that might increase representation of People of Color within recovery housing, especially among Black/African American people. There is also a lack of research that examines recovery home access among different populations, and how various populations pay for recovery housing. The majority of the studies in this category are quantitative in nature, and qualitative studies that capture the experiences and specific recovery housing needs across various populations, especially those who are often underrepresented in the recovery housing setting or those who have lower retention rates, are few. Since one of the key features of recovery housing is adoption of the social model of recovery, and if building solid, trusting relationships is important for long-term recovery, then the paucity of articles that investigate the unique needs across diverse populations is noteworthy.

### MOUD

Historically, most recovery residences have been abstinence-based, which had prohibited the use of any substances and considered medications like MOUD within this prohibited class. However, recent evidence indicates greater acceptance of MOUD and is working toward redefining what it means to be in recovery.

A growing body of literature addresses RH and MOUD treatment, but there is a gap in research on these combined concepts. The field has come to accept the value of RH and MOUD independently, but more research is needed on the impact of combined treatment. Furthermore, more research is needed to discover implementation results across the US. Current research appears to vary widely across geographic locations, with acceptance rates dependent on individual program values instead of evidence-based support. Current research outlines stigma from within and outside of the recovery community as a growing concern. Findings suggest that educational training has been beneficial in reducing that stigma and increasing acceptance rates. However, studies have yet to be conducted to find the most effective means of intervention training across communities.

### PRSS

Though RH inherently incorporates the notion of peer support, especially Level 1 programs, JEAP CB questions pertaining to explicitly incorporating peer recovery support services remains unanswered. Future research may benefit from analyzing the different styles of peer recovery support implementation.

### Economic impacts

Our search yielded only one study answering the CBs’ question regarding broad economic impacts of RH, which took place in one major US city and was published in 1989. Though the paper did find substantial decreases in use of other services (e.g., short-term detoxification), more work is needed. Thus, an important future line of inquiry will evaluate how RH might lead to changes in economic factors like the use of healthcare and Medicaid dollars, child welfare system costs, etc.

### Limitations

Several limitations for the current study should be considered. First, under the guidance of the JEAP CBs, NARR Level IV recovery houses and therapeutic communities were excluded from the study. Although this reflects a choice to emphasize an under-researched area of recovery, it means our findings do not reflect the full range of available recovery housing options. To this end, the relative robustness of research surrounding Level IV recovery houses and therapeutic communities may merit an independent review.

Additionally, the methodological rigor of the included studies varied greatly. Of the 86 quantitative studies identified, only three utilized randomized controlled trials which limits our ability to make causal inferences about the relationships between variables studied within RH and their related outcomes. As noted in the demographic section, many studies fail to report detailed demographics, limiting our ability to understand whether the current evidence base is applicable to the diverse population who stands to benefit from this area research.

## Conclusion

This project combined community-based participatory research and scoping study methodologies to examine whether research answers questions service recipients and practitioners pose. The JEAP Initiative performed CBPR to develop a set of research priorities regarding recovery support services, including recovery housing. This process resulted in 12 topical areas, each with a problem statement and set of testable research questions. The current study used a scoping study methodology to examine whether the literature answers questions within the RH category. Results indicate 111 studies pertaining to the research questions, though many more studies explore RH more broadly. Though research has discovered some guidance regarding “key ingredients” for RH and resident success, as well as serving various populations (especially marginalized identities), more work is required to obtain guidance for the most effective and efficient provision of this vital service. Particularly, studies with a high degree of rigor are called for, and the field would benefit from better reporting. Finally, detailed theoretical conceptualization would provide a framework for current and future work.

## Data Availability

The original contributions presented in the study are included in the article/Supplementary material, further inquiries can be directed to the corresponding author.

## Footnotes

^1^Note that the term “community board” is used intentionally, as opposed to the traditional “community advisory board.” The JEAP Initiative team considered these boards to be an integral part of the research process as co-producers, not “advisers.”

^2^A note here that CB members were not “participants” in the traditional research sense. They were collaborators in the research process.

^3^Note that a correction was published in 2018 correcting the author list (84).

## Supplementary material

SUPPLEMENTARY DATA SHEET 1 List of all RH studies included in analysis.

SUPPLEMENTARY DATA SHEET 2 List of additional RH studies not relevant to analysis.

SUPPLEMENTARY DATA SHEET 3 Table of demographics available in studies included in analysis.

SUPPLEMENTARY DATA SHEET 4 Search criteria used in study search.
